# Variation and trends in reasons for knee replacement revision: a multi-registry study of revision burden

**DOI:** 10.1080/17453674.2020.1853340

**Published:** 2020-12-02

**Authors:** Peter L Lewis, Otto Robertsson, Stephan E Graves, Elizabeth W Paxton, Heather A Prentice, Annette W-Dahl

**Affiliations:** a Australian Orthopaedic Association National Joint Replacement Registry, Adelaide, Australia;; b Swedish Knee Arthroplasty Register, Lund, Sweden;;; c Surgical Outcomes and Analysis, Kaiser Permanente, San Diego, CA, USA;; d Lund University, Faculty of Medicine, Clinical Science Lund, Department of Orthopedics, Lund, Sweden

## Abstract

Background and purpose — Studies describing time-related change in reasons for knee replacement revision have been limited to single regions or institutions, commonly analyze only 1st revisions, and may not reflect true caseloads or findings from other areas. We used revision procedure data from 3 arthroplasty registries to determine trends and differences in knee replacement revision diagnoses.

Patients and methods — We obtained aggregated data for 78,151 revision knee replacement procedures recorded by the Swedish Knee Arthroplasty Register (SKAR), the Australian Orthopaedic Association National Joint Replacement Registry (AOANJRR), and the Kaiser Permanente Joint Replacement Registry (KPJRR) for the period 2003–2017. Equivalent diagnosis groups were created. We calculated the annual proportions of the most common reasons for revision.

Results — Infection, loosening, and instability were among the 5 most common reasons for revision but magnitude and ranking varied between registries. Over time there were increases in proportions of revisions for infection and decreases in revisions for wear. There were inconsistent proportions and trends for the other reasons for revision. The incidence of revision for infection showed a uniform increase.

Interpretation — Despite some differences in terminology, comparison of registry-recorded revision diagnoses is possible, but defining a single reason for revision is not always clear-cut. There were common increases in revision for infection and decreases in revision for wear, but variable changes in other categories. This may reflect regional practice differences and therefore generalizability of studies regarding reasons for revision is unwise.

Although the survivorship of knee arthroplasty has improved over the last 15 years, the increased volume of primary knee replacement has led to growing numbers of revision procedures (Kumar et al. 2015, Patel et al. 2015). A prior study we undertook outlined changes in the volume and incidence of revision rates in Sweden, Australia, and the Kaiser Permanente registry from the USA (Lewis et al. 2020b).

Factors influencing revision change with time. Patient factors may affect the rate of primary procedures, such as rising patient and surgeon acceptance of knee replacement (Hamilton et al. 2015), increasing rates of osteoarthritis (Hunter and Bierma-Zeinstra 2019), growing use in younger patients (Leyland et al. 2016, Karas et al. 2019), and also survivorship, such as longer life expectancy, increasing obesity, and higher physical activity of those receiving a replacement (Hamilton et al. 2015). In addition, prosthesis designs change to improve perceived shortcomings such as wear, instability, and patellofemoral pain and tracking (Lewis et al. 2020a). Methods to improve surgical precision, such as computer navigation (Jones and Jerabek 2018), image-derived instrumentation (Kizaki et al. 2019), and robotic assistance (Jacofsky et al. 2016) may decrease revision requirements (Price et al. 2018)

These changing factors alter the reasons for revision. Previous studies observed a decrease in revisions for wear and loosening (Sharkey et al. 2014, Thiele et al. 2015), and related this to improved prosthesis design and materials. Other studies note infection is now the most common reason for revision (Koh et al. 2017, Postler et al. 2018). Studies of changing knee replacement failure modes are limited by being derived from single institutions or regions and may not accurately reflect what is occurring elsewhere (Sharkey et al. 2014, Thiele et al. 2015, Dyrhovden et al. 2017, Koh et al. 2017, Lum et al. 2018, Postler et al. 2018). Additionally, these studies do not show the true revision burden as they are restricted to 1st revision procedures, or only revisions of previous total knee replacements (TKR), and do not include revisions of partial knee replacement procedures.

Combining registry data can be difficult due to inconsistency in the definition of revision (Liebs et al. 2015), and lack of consensus in defining modes of failure, with different terminologies used (Niinimaki 2015, Siqueira et al. 2015). Some have attempted to overcome this by defining equivalent diagnoses (Havelin et al. 2011, Paxton et al. 2011, Rasmussen et al. 2016).

We determined variations and trends in reasons for knee replacement revision using data on all knee arthroplasty revision procedures from the national registries of Sweden and Australia and the institutional registry of Kaiser Permanente in the USA by using equivalent diagnosis groups (Table 1, see Supplementary data).

## Patients and methods

We obtained data for the period January 1, 2003 until December 31, 2017 for all revision knee replacement procedures recorded in the Swedish Knee Arthroplasty Register (SKAR), Australian Orthopaedic Association National Joint Replacement Registry (AOANJRR), and the Kaiser Permanente Joint Replacement Registry (KPJRR).

Revision knee replacements included all revision procedures of a previous replacement where 1 or more components were added, removed, or exchanged, regardless of whether this was the 2nd or subsequent procedure in chronology. Revisions of all types of knee replacement were included irrespective of whether the arthroplasty was a partial or total knee replacement. Where knee revisions were bilateral, both knees were included and recorded separately. The capture rate or completeness of these registries exceeds 95% and loss to follow-up was less than 8% over the study period. Validation and quality control methods of these registries have been published (Paxton et al. 2010, Robertsson et al. 2014, AOANJRR 2019).

In all registries the reason for revision was determined from the revision diagnosis selected by the surgeon at the time of the revision procedure from a predetermined list, or specifically added. Multiple reasons could be listed. In Sweden all operative reports were methodically read and from these the primary reason for revision was interpreted by registry staff. In the AOANJRR and KPJRR, when multiple reasons for revision were recorded, a diagnosis hierarchy was used to determine the most important reason for revision. In this study only one reason for revision was permitted for each revision procedure.

We included 78,151 revision knee replacement procedures. The SKAR contributed 12,612 revision procedures, the AOANJRR 53,853 revisions, and the KPJRR 11,686 revisions.

Using the categories from the SKAR as a basis, a table of equivalent diagnoses was created. For each registry the reasons for revision were then reclassified according to the “harmonized diagnosis” category.

### Statistics

Aggregated data regarding procedure numbers, patient age, and sex were obtained for each registry (Table 2, see Supplementary data). After categorization using the equivalent diagnosis method, the number of revisions for each of the 10 most common reasons was determined and the remainder classed as “other” (Table 3, see Supplementary data). The “other” category also included a small percentage of missing data (1.1% or 137 procedures) from Sweden. The “other” group from the KPJRR contained those with a recorded diagnosis of “failed TKR,” which contributed between 3.3% and 12% of all revisions each year.

For all registries the annual proportions of each harmonized revision diagnosis were calculated. For further analysis of revision for infection, the incidence per 100,000 was calculated from population data obtained from Statistics Sweden and the Australian Bureau of Statistics, as well as the yearly active membership numbers from Kaiser Permanente.

### Ethics, funding, and conflicts of interest

Ethics approval covering the SKAR data use was issued by the Ethics Board of Lund University (LU20-02). The AOANJRR is a declared Commonwealth of Australia Quality Assurance Activity under section 124X of the Health Insurance Act, 1973. All AOANJRR studies are conducted in accordance with the ethical principles of research (Helsinki Declaration II). Approval for inclusion of data from the Kaiser Permanente Joint Replacement Registry Institutional Review Board r(#5488) was granted on November 15, 2018.

There was no funding. There are no conflicts of interest.

## Results

**Figure 1.**
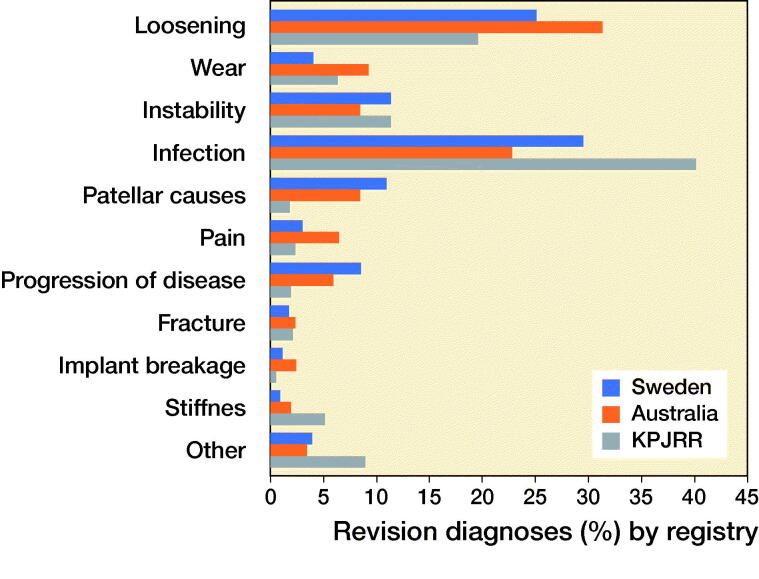
Overall revision diagnoses shown as a proportion for each registry.

Considering all revisions during the entire time period, infection was the most frequent revision diagnosis in the SKAR and KPJRR while loosening was most common in the AOANJRR. Instability, patellar causes, progression of disease, wear, and pain showed variable proportions across the registries (Figure 1.)

The number of revisions and yearly proportions for each of the 10 most common reasons for revision are given in Table 3 (see Supplementary data) and a graphical representation of the proportions to highlight trends is shown in Figure 2.

Figure 2.Yearly proportions of knee replacement revision recorded in the SKAR, the AOANJRR, and the KPJRR, respectively.

**Figure:**
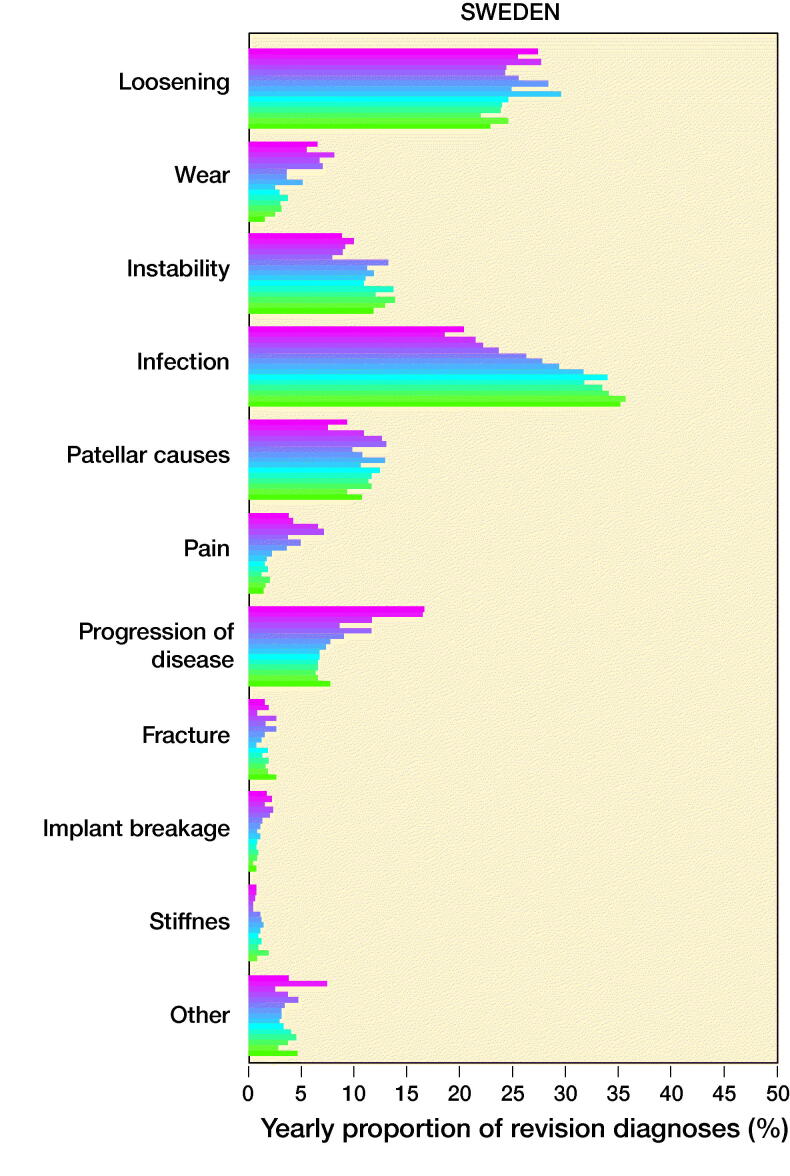

**Figure:**
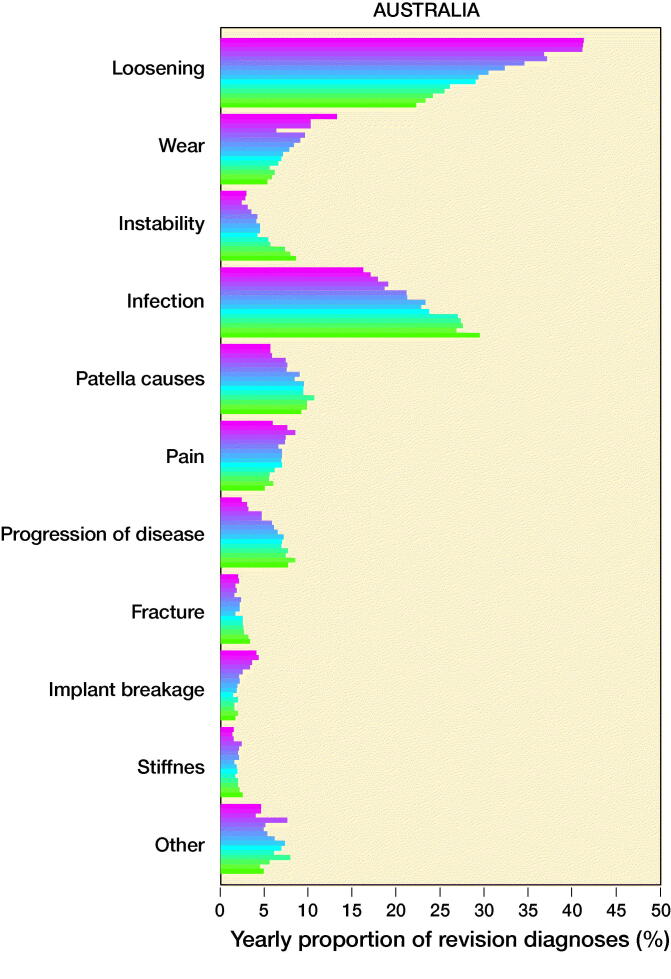

**Figure:**
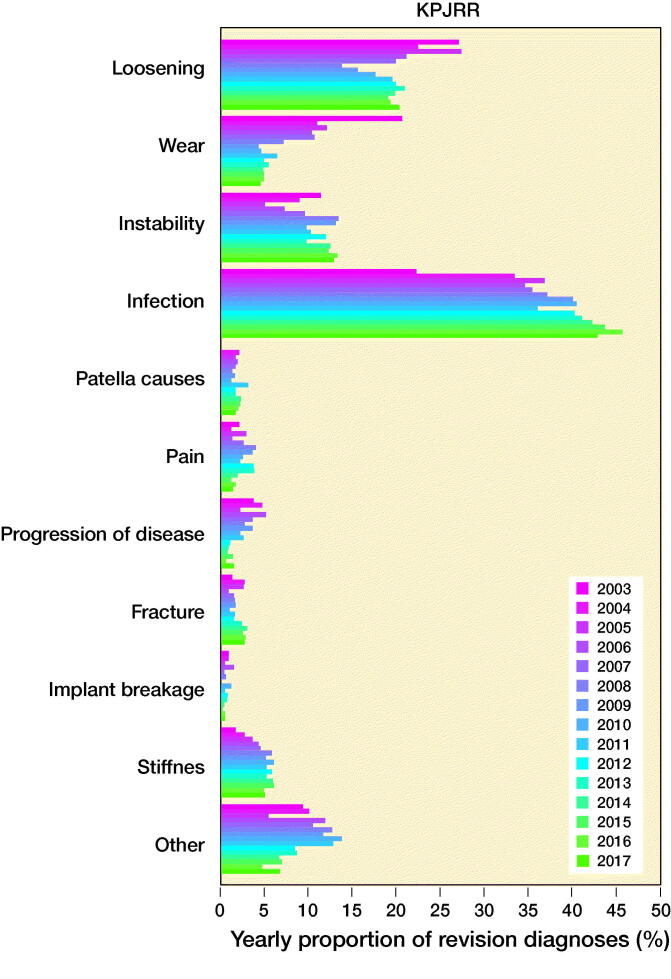

**Figure 3.**
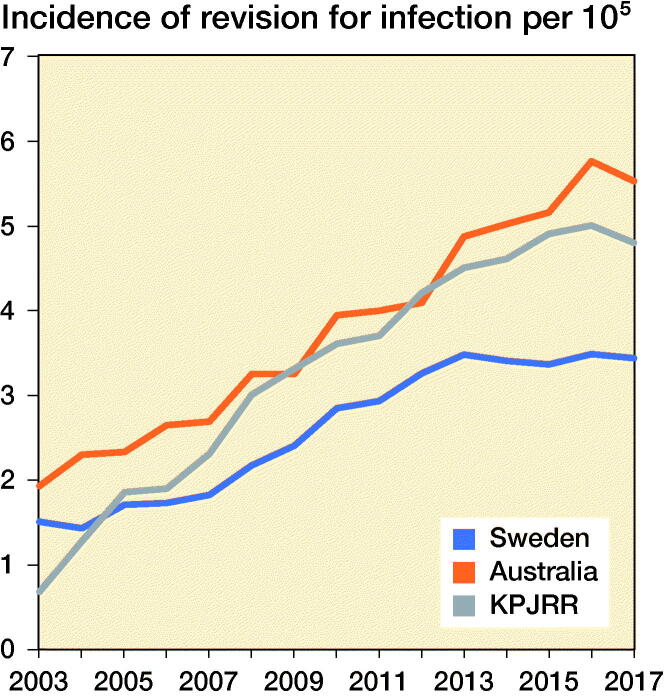
Yearly incidence of revision knee replacement for infection per 100,000 population for the SKAR, AOANJRR, and KPJRR.

In all registries, there was an increase in the proportion of revisions for infection through the study period rising from 20%, 16%, and 22% in the Swedish, Australian, and KP registries in 2003 to 35%, 30%, and 43% in 2017, respectively. To determine whether this was a true rise, not just a proportionate increase, the yearly incidence of revision procedures for infection was calculated. This also increased in all registries (Figure 3.) Revision for loosening fell from 41% in 2003 to 13% in 2017 in the AOANJRR but a smaller decline was seen in the SKAR (27% to 23%), while the proportion in the KPJRR fell from 27% in 2003 to 14% 2008 but then rose and remained around 20% from 2011 to 2017. There was a universal decrease in revisions for wear with the proportions declining from 6.5% to 1.5% in Sweden, 13% to 5.3% in Australia, and 21% to 4.8% in the KPJRR. Instability as a revision diagnosis showed a trend for increase in Sweden and Australia, but fluctuated in the KPJRR. Revisions for patellar reasons contributed to a higher proportion of revisions in Sweden than Australia, showing a modest increase in these 2 countries while this diagnosis was infrequent in the KPJRR. Stiffness contributed proportionally more as a revision diagnosis in the KPJRR, where this reason showed a small increase with time. There was a general tendency for fewer revisions for pain throughout all registries toward the end of the time period. Progression of disease decreased over time in both Sweden and the KPJRR while it increased in Australia as a reason for revision. Fracture and implant breakage were uncommon causes of revision in all registries.

## Discussion

We have previously shown a decrease in all-cause revision rates in all 3 of these registries, but the reasons for revision were not studied (Lewis et al. 2020b). In the present study, when considering the entire study period, infection, loosening, and instability were among the 5 most common reasons for revision in all 3 registries; however, ranking and proportions of these varied. Over time, reasons for knee replacement revision changed, and while there were some similarities in rising proportions of revisions for infection, and decreasing proportions for wear, there were also differences between registries in 8 of the 10 most common revision reasons. These findings suggest revision reasons are partially dependent on factors specific to each healthcare system, and while variation in prosthesis use may be a major cause, analysis of this aspect is the subject of a further study.

A limitation of this study is that categorizing revision diagnoses can be subjective. While many diagnoses are self-evident, in a knee replacement with pronounced wear, loosening, instability, and prosthesis breakage it can be difficult to determine which is the main cause of failure. This choice may vary between surgeons. There may be differences in interpretation: where one surgeon may nominate “progression of disease” as the reason for revision, another may record “patella erosion” for the same clinical findings. These interpretive differences can exist both within and between registries. A technique to limit the effect of this would be to correlate the revision diagnosis with the revision procedure.

Using the method of equivalent diagnoses, we created a “cross-walk” between reported reasons for revision in each of the registries. Most categorizing of revision reasons is straightforward but in a few instances creation of a format to compare registry results is also open to subjectivity. For example, the diagnosis of “inflammatory arthritis” in the KPJRR has been considered as “progression of disease” but may be the equivalent to the AOANJRR diagnosis of “synovitis,” which has been classed as “other.” While malalignment is a revision diagnosis in the AOANJRR, neither the SKAR nor the KPJRR record this specific diagnosis separately, and therefore these are included in the “other” category. Registries may also have “systematic” differences in ranking of relative importance where more than 1 diagnosis is reported. These classification and ranking issues are likely to have only a small effect on the overall results.

A further limitation is that while we included all knee revision procedures to compare revision burdens and changing reasons for revision with time, we could not determine whether these changes relate to the first or subsequent revisions. However, previous registry analyses have shown that 60–85% of annual revisions are first revisions (AOANJRR 2019). There was a universal increase in proportion and yearly incidence of revisions for infection in the 3 registries studied. The reason for this worrying widespread increase is not clear, but is consistent with the findings of others (Sharkey et al. 2014, Dyrhovden et al. 2017, Koh et al. 2017). It has been suggested that debridement, antibiotics, and implant retention with only polyethylene insert exchange (DAIR) is being increasingly and more aggressively used for the treatment of periprosthetic infection (Kunutsor et al. 2018).

Increases in revisions for infection are even more concerning as registries under-report infection, particularly missing non-revision episodes of treatment that do not have a prosthetic component removed or replaced (Witsø 2015, Zhu et al. 2016). In the AOANJRR, where the reason for revision is recorded at the time of operation, there may be under-reporting of infection where delayed culture results are returned as positive and, similarly, there may be a small proportion of over-reporting where a suspicion of infection is not supported by microbiological results. This type of inaccuracy would be lower in the SKAR and KPJRR as these registries can postoperatively modify the recorded diagnosis of infection on the basis of microbiological results (SKAR 2019).

Revisions for wear decreased in all 3 registries, which is also a finding reported by others (Le et al. 2014, Sharkey et al. 2014, Thiele et al. 2015). Proposed reasons for this decrease are improvements in polyethylene by modified sterilization and packaging methods (Faris et al. 2006), increased use of highly cross-linked polyethylene (de Steiger et al. 2015), increased bearing conformity (Zhang et al. 2019), altered knee kinematics with femoral component design changes (Gilbert et al. 2014), or decreased tibial baseplate roughness and improved polyethylene locking mechanisms (Sisko et al. 2017).

Loosening decreased as a reason for revision in both the SKAR and AOANJRR but remained unchanged in the KPJRR. The SKAR can determine which components have loosened from the operative records, but in the other 2 registries this is not possible. While an impression may be obtained by correlation with the components changed in the revision procedures, this may not be precise as, for example, if tibial loosening alone is present, both major components may be revised to allow for increased stability in the revision prosthesis configuration. Late loosening is thought to be related to wear and its consequence of osteolysis (Holt et al. 2007) and would be expected to decrease as polyethylene wear decreases. Early loosening, in contrast, most likely relates to a lack of initial fixation and is greater where cementless prostheses are used with the intent of biological fixation (Aprato et al. 2016). While our study did not explore prostheses attributes, the inter-registry differences in loosening may relate to the proportional use of cementless implants or factors such as different bone cements and cementing techniques or types of polyethylene inserts used.

The Swedish and Australian registries showed an increase in proportion of revision for instability. While this finding supports previous reports (Thiele et al. 2015, Dyrhovden et al. 2017), it contrasts with another where a decrease has been shown (Sharkey et al. 2014). An explanation for this change could be an increase in recognition of instability, where revisions that were once diagnosed as pain of unknown origin have increasingly been interpreted as pain due to instability (Firestone and Eberle 2006, Grayson et al. 2016). Another possibility is the development of new knowledge, with the dissemination and acceptance of the concept of mid-flexion instability during the study period (Ramappa 2015, Longo et al. 2020). There may also be a link between instability revisions and the use of posterior cruciate substituting prostheses (Hino et al. 2013).

Patellar causes for revision made up a consistently higher proportion of revisions in Sweden, followed by Australia and then the KPJRR. While revisions in this category predominantly involve secondary insertion of a patellar component in a previously un-resurfaced patella and much of this difference may relate to the use of patellar components at the time of primary surgery, it also includes patellar component revisions and even patellectomy. In 2018 in Sweden there was a 3% rate of primary patellar component use (SKAR 2019), in Australia the rate of use has climbed from 42% in 2005 to 69% in 2018 (AOANJRR 2019), while in the KPJRR patellar component use has been reported at 98% (Paxton et al. 2011). Leaving the patella unresurfaced allows the potential need for a secondary resurfacing procedure. Additionally, there may be differences relating to the prostheses used with respect to generation of anterior knee pain or other patellar complications such as mal-tracking.

While there were no consistent trends in revision for progression of disease or for pain, these 2 categories are more difficult to understand. Revision for progression of disease was higher in Sweden than in the other 2 registries, and may, in part, be explained by the possible inclusion of patellar erosion or patellar degenerative change of an un-resurfaced patella as diagnoses in this category. The proportion of knees revised for progression of disease in Sweden decreased with time, and may mirror the fall in proportional use of unicompartmental knee replacement (from 13% of primary knee replacement in 2003 to 9% in 2017) (Lewis et al. 2020b) . However, these factors cannot explain the increase in revision for progression of disease in Australia, where there has been a decrease in use of unicompartmental knee replacement (from 15% of primary knee replacement in 2003 to 6% in 2017) with an increase in patellar component use (from 41% of primary TKR in 2005 to 67% in 2017) (AOANJRR 2019). Similarly, this cannot explain the decline in the KPJRR where unicompartmental knee use and patellar resurfacing remained constant (at 4% and 98% respectively) (Lewis et al. 2020b, Paxton et al. 2011). (The annual procedure numbers of partial and total knee replacement for each registry have been described in our previous paper—Lewis et al. 2020b). Other covert factors, such as the inclusion of revisions of knee replacements from the time prior to the commencement of this study where the proportions of unicompartmental or patellar prosthesis use are unknown, may contribute to these findings.

The revision diagnoses of fracture, stiffness, and component breakage occurred infrequently. Fracture as a reason for revision showed a small increase, which is possibly related to a globally ageing and more osteoporotic knee replacement population (Johnson et al. 2019). Revision for fracture would understate the frequency of periprosthetic fracture, as many of these are treated by means other than revision, such as fracture fixation alone. Stiffness or true arthrofibrosis is rare, and there can be cultural differences in patients, and possibly even their surgeons, proceeding to revision surgery for this reason (Springer et al. 2012). Similar to fracture, registry data does not reflect the true incidence of stiffness, as non-revision treatment methods, such as manipulation under anesthetic, are not included. A decline in implant breakage may reflect improved component durability.

Of concern is the “other” diagnosis category from the KPJRR, which included a diagnosis of “failed TKR.” The true reason for revision in these procedures is unclear, but the proportion in the “other” group decreased over the study period, indicating improving precision of revision diagnosis records in this registry. The influence of this is difficult to determine.

In conclusion, we have shown that despite some differences in terminology it is possible to compare registry data regarding reasons for revision. Defining a single reason for knee replacement revision is not always clear-cut. While infection, loosening, and instability are within the 5 most common reasons for revision for all 3 registries studied, their magnitude and ranking varied through the period. There were consistent increases in revision for infection, and decreases in revision for wear, but variable changes in other categories. Findings from the 3 registries studied differed, which may reflect regional differences in patient, prosthesis, or technique characteristics, and further study is required to define these practice variations. Widespread generalizability of studies regarding reasons for knee replacement revision may not be prudent. There may also be a place for defining the revision diagnoses by an international consensus, in the method Kalson et al. (2016) used for arthrofibrosis, which would give clarity, consistency, and better understanding of this area.

## Supplementary Material

Supplemental Material

## Supplementary data

Tables 1–3 are available as supplementary data in the online version of this article, http://dx.doi.org/10.1080/17453674.2020.1853340
