# Supplementary material for: Variation and trends in reasons for knee replacement revision: a multi-registry study of revision burden
Source: Acta Orthop. 2020 Dec 2;92(2):182–8. doi: 10.1080/17453674.2020.1853340 (PMC8159200; doi:10.1080/17453674.2020.1853340)
Supplement: Supplemental Material [file IORT_A_1853340_SM1040.pdf]

Supplementary data

Table 1. Table of equivalent diagnoses for each registry

Harmonized diagnosis	Sweden diagnoses	Australian diagnoses	KP diagnoses
1. Loosening	Loosening	Loosening	Aseptic loosening<br>Ingrowth failure
2. Wear	Implant wear	Wear tibial insert<br>Lysis<br>Metal related pathology<br>Wear tibia<br>Wear patella<br>Wear femur	Poly liner wear<br>Osteolysis<br>Implant wear
3. Instability	Instability<br>Dislocated polyethylene	Instability<br>Bearing dislocation<br>Prothesis dislocation	Instability
4. Infection	Infection	Infection	Infection<br>wound drainage
5. Patellar causes	Patellar causes	Patellofemoral pain<br>Patellar erosion<br>Patellar maltracking	Patellofemoral joint malfunction<br>Failed extensor mechanism<br>Patellar causes
6. Pain	Pain	Pain	Pain
7. Progression of disease	Progress of disease	Progression of disease	Osteoarthritis<br>Inflammatory arthritis<br>Old rheumatoid<br>Posttraumatic arthritis
8. Fracture	Bone fracture	Fracture	Fracture
9. Implant breakage	Implant fracture	Implant breakage tibial insert<br>Implant breakage tibia<br>Implant breakage patella<br>Implant breakage femur	Component fracture/breakage
10. Stiffness	Stiffness	Arthrofibrosis	Arthrofibrosis/stiffness
11. Other	Other<br>Tumor<br>Surgical error<br>Missing	Malalignment<br>Incorrect sizing<br>Synovitis<br>Osteonecrosis<br>Tumor<br>Heterotopic bone<br>Incorrect side<br>Other	Failed TKR<br>Failed UKA<br>Failed Unispacer<br>AVN<br>Synovial impingement<br>Hematoma<br>Other
12. Excluded (non-revision procedures)	Gangrene<br>Cement/free body		Wound dehiscence<br>Failed ORIF

Table 2. Yearly procedure numbers, mean ages, and percentage of females for revision knee replacement 2003– 2017 by registry

Registry	2003	2004	2005	2006	2007	2008	2009	2010	2011	2012	2013	2014	2015	2016	2017
Sweden															
Revisions, n	657	686	715	698	698	757	801	904	870	903	1,043	980	962	966	972
Mean age	70.7	70.5	70.5	69.9	69.9	69.8	69.2	68.7	68.5	69.4	68.8	68.3	68.5	69.4	69.9
Female sex (%)	58.2	61.0	59.2	61.5	57.5	54.0	61.1	56.6	56.1	55.5	55.5	51.9	54.2	54.6	53.2
Australia															
Revisions, n	2,314	2,663	2,721	2,826	2,994	3,250	3,294	3,716	3,984	3,910	4,173	4,301	4,447	4,559	4,791
Mean age	69.9	68.8	69.5	69	69.1	68.7	68.9	68.4	68.6	68.5	68.7	68.6	68.2	68.9	68.9
Female sex (%)	51.6	51.8	50.5	51.5	52.7	51.9	50.8	52.2	50.7	52.3	51.2	52.4	51.3	50	51.1
Kaiser Permanente															
Revisions, n	247	338	423	473	556	704	706	780	908	947	992	1,051	1,148	1,177	1,236
Mean age	68.0	67.7	67.5	66.2	67.9	66.6	66.5	67.4	66.6	66.8	67.1	67.2	67.1	67.4	67.7
Female sex (%)	47.4	53.9	53.2	55.6	54.0	54.1	58.4	55.5	53.0	58.6	52.8	56.0	56.5	52.8	54.6

Table 3a. Yearly number and proportions of reasons for revision — Sweden

Reason for revision	2003	2004	2005	2006	2007	2008	2009	2010	2011	2012	2013	2014	2015	2016	2017
Loosening, n	180	176	189	171	170	194	228	225	258	224	251	235	213	238	224
%	*27.4*	*25.5*	*27.7*	*24.4*	*24.3*	*25.6*	*28.4*	*24.9*	*29.6*	*24.6*	*24.0*	*23.9*	*22.0*	*24.6*	*22.9*
Wear, n	43	38	58	47	49	27	29	46	22	26	39	30	30	24	15
%	*6.5*	*5.5*	*8.1*	*6.7*	*7.0*	*3.6*	*3.6*	*5.1*	*2.5*	*2.9*	*3.7*	*3.0*	*3.1*	*2.5*	*1.5*
Instability, n	58	69	65	62	55	100	90	108	97	99	144	118	134	125	116
%	*8.8*	*10.0*	*9.1*	*8.9*	*7.9*	*13.2*	*11.2*	*11.9*	*11.1*	*10.9*	*13.7*	*12.0*	*13.9*	*12.9*	*11.8*
Infection, n	135	128	154	156	166	199	223	266	276	309	333	330	329	345	345
%	*20.4*	*18.6*	*21.5*	*22.2*	*23.7*	*26.3*	*27.8*	*29.4*	*31.7*	*34.0*	*31.8*	*33.5*	*34.1*	*35.7*	*35.2*
Patellar causes, n	61	52	78	88	91	74	87	117	92	113	122	111	112	90	105
%	*9.3*	*7.5*	*10.9*	*12.6*	*13.0*	*9.8*	*10.8*	*12.9*	*10.6*	*12.4*	*11.6*	*11.3*	*11.6*	*9.3*	*10.7*
Pain, n	25	29	47	50	26	37	29	20	15	14	19	12	19	15	14
%	*3.8*	*4.2*	*6.6*	*7.1*	*3.7*	*4.9*	*3.6*	*2.2*	*1.7*	*1.5*	*1.8*	*1.2*	*2.0*	*1.6*	*1.4*
Progr. of disease, n	109	114	84	60	81	68	62	66	59	61	68	65	61	64	75
%	*16.6*	*16.5*	*11.7*	*8.6*	*11.6*	*9.0*	*7.7*	*7.3*	*6.7*	*6.7*	*6.6*	*6.6*	*6.3*	*6.6*	*7.7*
Fracture	10	13	6	18	11	20	12	11	6	16	14	19	15	17	25
%	*1.5*	*1.9*	*0.8*	*2.6*	*1.6*	*2.6*	*1.5*	*1.2*	*0.7*	*1.8*	*1.3*	*1.9*	*1.6*	*1.8*	*2.6*
Implant breakage, n	11	15	11	16	14	10	9	7	10	7	7	9	8	4	7
%	*1.7*	*2.2*	*1.5*	*2.3*	*2.0*	*1.3*	*1.1*	*0.8*	*1.1*	*0.8*	*0.7*	*0.9*	*0.8*	*0.4*	*0.7*
Stiffness, n	0	5	5	4	3	3	9	11	12	10	9	12	9	18	8
%	*0*	*0.7*	*0.7*	*0.6*	*0.4*	*0.4*	*1.1*	*1.2*	*1.4*	*1.1*	*0.9*	*1.2*	*0.9*	*1.9*	*0.8*
Other, n	25	47	18	26	32	25	23	27	23	24	37	39	32	26	38
%	*3.8*	*7.4*	*2.5*	*3.7*	*4.7*	*3.4*	*3.1*	*3.1*	*2.9*	*3.3*	*4.0*	*4.5*	*3.7*	*2.8*	*4.6*
Total, n	657	686	715	698	698	757	801	904	870	903	1,043	980	962	966	972

Table 3b. Yearly number and proportions of reasons for revision — Australia

Reason for revision	2003	2004	2005	2006	2007	2008	2009	2010	2011	2012	2013	2014	2015	2016	2017
Loosening, n	957	1,099	1,122	1,043	1,113	1,124	1,067	1,133	1,145	1,133	1,091	1,097	1,078	1,070	1,068
%	*41.4*	*41.3*	*41.2*	*36.9*	*37.2*	*34.6*	*32.4*	*30.5*	*29.4*	*29*	*26.1*	*25.5*	*24.2*	*23.4*	*22.3*
Wear, n	307	274	281	179	288	295	274	290	275	269	277	240	278	263	254
%	*13.3*	*10.3*	*10.3*	*6.3*	*9.6*	*9.1*	*8.3*	*7.8*	*7.1*	*6.9*	*6.6*	*5.6*	*6.2*	*5.8*	*5.3*
Instability, n	68	75	66	87	104	136	135	168	175	164	227	145	326	361	413
%	*2.9*	*2.8*	*2.4*	*3.1*	*3.5*	*4.2*	*4.1*	*4.5*	*4.5*	*4.2*	*5.4*	*5.7*	*7.3*	*7.9*	*8.6*
Infection	378	456	468	540	559	688	701	869	892	930	1,127	1,179	1,227	1,228	1,413
%	*16.3*	*17.1*	*17.9*	*19.1*	*18.7*	*21.2*	*21.3*	*23.4*	*22.9*	*23.8*	*27.0*	*27.4*	*27.6*	*26.9*	*29.5*
Patellar causes, n	129	150	156	207	229	244	295	312	368	369	392	462	436	436	442
%	*5.7*	*5.7*	*5.8*	*7.4*	*7.6*	*7.5*	*9.0*	*8.4*	*9.5*	*9.4*	*9.4*	*10.7*	*9.8*	*9.8*	*9.2*
Pain, n	137	202	230	209	218	213	232	260	269	275	257	239	246	275	241
%	*5.9*	*7.6*	*8.5*	*7.4*	*7.3*	*6.6*	*7.0*	*7.0*	*6.9*	*7.0*	*6.2*	*5.6*	*5.5*	*6.0*	*5.0*
Progr. of disease, n	55	80	88	133	141	187	201	242	281	274	290	330	328	386	367
%	*2.4*	*3.0*	*3.2*	*4.7*	*4.7*	*5.8*	*6.1*	*6.5*	*7.2*	*7.0*	*6.9*	*7.7*	*7.4*	*8.5*	*7.7*
Fracture, n	47	55	46	51	49	74	71	82	66	97	105	113	122	144	157
%	*2.0*	*2.1*	*1.7*	*1.8*	*1.6*	*2.3*	*2.2*	*2.2*	*1.7*	*2.5*	*2.5*	*2.6*	*2.7*	*3.2*	*3.3*
Implant breakage, n	94	115	97	92	76	67	73	70	69	56	82	68	70	90	82
%	*4.1*	*4.3*	*3.6*	*3.3*	*2.5*	*2.1*	*2.2*	*1.9*	*1.8*	*1.4*	*2.0*	*1.6*	*1.6*	*2.0*	*1.7*
Stiffness, n	35	35	41	69	63	64	70	59	70	73	70	88	88	99	120
%	*1.5*	*1.3*	*1.5*	*2.4*	*2.1*	*2.0*	*2.1*	*1.6*	*1.8*	*1.9*	*1.7*	*2.0*	*2.0*	*2.2*	*2.5*
Other, n	107	122	108	216	154	158	175	231	284	270	255	340	248	207	234
%	*4.6*	*4.6*	*4.0*	*7.6*	*5.1*	*4.9*	*5.3*	*6.2*	*7.3*	*6.9*	*6.1*	*7.9*	*5.6*	*4.5*	*4.9*
Total, n	2,314	2,663	2,721	2,826	2,994	3,250	3,294	3,716	3894	3,910	4,173	4,301	4,447	4,559	4,791

Table 3c. Yearly number and proportions of reasons for revision — Kaiser Permanente

Reason for revision	2003	2004	2005	2006	2007	2008	2009	2010	2011	2012	2013	2014	2015	2016	2017
Loosening, n	67	76	116	100	111	97	110	137	177	189	208	208	218	227	251
%	*27.1*	*22.5*	*27.4*	*21.1*	*20.0*	*13.8*	*15.6*	*17.6*	*19.5*	*20.0*	*21.0*	*19.8*	*19.0*	*19.3*	*20.3*
Wear, n	51	37	51	49	59	50	30	36	58	46	54	50	56	57	56
%	*20.7*	*11.0*	*12.1*	*10.4*	*10.6*	*7.1*	*4.3*	*4.6*	*6.4*	*4.9*	*5.4*	*4.8*	*4.9*	*4.8*	*4.5*
Instability, n	28	30	21	34	53	94	92	76	93	113	96	131	141	156	159
%	*11.3*	*8.9*	*5.0*	*7.2*	*9.5*	*13.4*	*13.0*	*9.7*	*10.2*	*11.9*	*9.7*	*12.5*	*12.3*	*13.3*	*12.9*
Infection, n	55	113	156	164	197	262	283	316	328	382	408	445	502	539	531
%	*22.3*	*33.4*	*36.9*	*34.7*	*35.4*	*37.2*	*40.1*	*40.5*	*36.1*	*40.3*	*41.1*	*42.3*	*43.7*	*45.8*	*43.0*
Patellar causes, n	0	7	7	9	9	9	11	9	28	16	16	24	25	23	21
%	*0.0*	*2.1*	*1.7*	*1.9*	*1.6*	*1.3*	*1.6*	*1.2*	*3.1*	*1.7*	*1.6*	*2.3*	*2.2*	*2.0*	*1.7*
Pain, n	5	4	12	6	14	28	25	19	20	35	37	19	13	20	17
%	*2.0*	*1.2*	*2.8*	*1.3*	*2.5*	*4.0*	*3.5*	*2.4*	*2.2*	*3.7*	*3.7*	*1.8*	*1.1*	*1.7*	*1.4*
Progr. of disease, n	9	16	9	24	20	19	25	17	23	10	8	8	15	6	18
%	*3.6*	*4.7*	*2.1*	*5.1*	*3.6*	*2.7*	*3.5*	*2.2*	*2.5*	*1.1*	*0.8*	*0.8*	*1.3*	*0.5*	*1.5*
Fracture, n	3	9	11	4	8	11	12	7	14	14	23	31	28	32	33
%	*1.2*	*2.7*	*2.6*	*0.9*	*1.4*	*1.6*	*1.7*	*0.9*	*1.5*	*1.5*	*2.3*	*3.0*	*2.4*	*2.7*	*2.7*
Implant breakage, n	2	3	2	7	2	4	0	9	4	7	6	4	2	5	6
%	*0.8*	*0.9*	*0.5*	*1.5*	*0.4*	*0.6*	*0.0*	*1.2*	*0.4*	*0.7*	*0.6*	*0.4*	*0.2*	*0.4*	*0.5*
Stiffness, n	4	9	15	20	25	41	36	47	47	55	51	62	69	57	61
%	*1.6*	*2.7*	*3.6*	*4.2*	*4.5*	*5.8*	*5.1*	*6.0*	*5.2*	*5.8*	*5.1*	*5.9*	*6.0*	*4.8*	*4.9*
Other, n	23	34	23	56	58	89	82	107	116	80	85	69	79	55	83
%	*9.3*	*10.1*	*5.4*	*11.8*	*10.4*	*12.6*	*11.6*	*13.7*	*12.8*	*8.5*	*8.6*	*6.6*	*6.9*	*4.7*	*6.7*
Total, n	247	338	423	473	556	704	706	780	908	947	992	1,051	1,148	1,177	1,236
